# Could carotid artery calcifications and pulp stones be an alarm sign for diabetes mellitus? A retrospective observational study

**DOI:** 10.1186/s12902-025-02005-z

**Published:** 2025-08-04

**Authors:** Motahare Baghestani, Mohadese Faregh, Seyed Hossein Razavi, Fatemeh Owlia

**Affiliations:** 1 Department of Oral and Maxillofacial Radiology, School of Dentistry, Shahid Sadoughi University of Medical Sciences, Yazd, Iran; 2 Dentist, School of Dentistry, Shahid Sadoughi University of Medical Sciences, Yazd, Iran; 3 Department of Oral and Maxillofacial Medicine, School of Dentistry, Shahid Sadoughi University of Medical Sciences, Yazd, Iran

**Keywords:** Carotid artery atheroma, Diabetes mellitus, Panoramic radiography, Prevalence, Pulp stones

## Abstract

**Background:**

Carotid artery calcifications and pulpal stones are radiopaque findings that may be found accidentally in panoramic views. The risk of affliction with atherosclerotic events in diabetic patients with dystrophic calcification is questionable. This study aimed to investigate the relative frequency of carotid calcifications and pulp stones in panoramic radiographs of diabetic patients.

**Methods:**

This retrospective observational study employed a convenience sampling method involving 107 diabetic patients. For comparison, 300 panoramic views from individuals estimated to be healthy in the community were included. The samples were randomly selected from the statistical population using a random numbers table. Data analysis was conducted using descriptive statistics, including mean and standard deviation, as well as analytical tests such as the chi-square test, all performed with SPSS 17 software (Chicago, USA).

**Results:**

In this study, panoramic radiographs of 107 diabetic patients (67 women and 40 men) and 300 healthy individuals (196 women and 104 men) were evaluated. The age range was 25 to 64 years, with a mean age of 49.7. The frequency of carotid artery calcification was 42 (14%) in healthy individuals and 44 (41.1%) in diabetic patients. Furthermore, the relative frequency of unilateral and bilateral carotid artery calcification in diabetic patients was significantly higher than in healthy subjects (*P* < 0.05). The Relative Risk (RR) of pulp stones in the diabetic patient group compared to healthy individuals was 1.8. With a 95% confidence interval, the relative risk ranged from 1.3 to 2.48, which was statistically significant (*P* < 0.05).

**Conclusions:**

Based on the findings, the frequency of carotid artery calcification and pulp stones was higher in diabetic patients. Panoramic radiographic screening in diabetic patients is useful for early detection of carotid artery calcification and timely referral of patients to endocrinologists to prevent adverse sequelae.

## Introduction

Heterotopic calcification occurs when calcium salts are placed in a disorganized environment. It includes three categories: metastatic, idiopathic, and dystrophic. When serum calcium and phosphorus levels are higher than normal limits, excess calcium is deposited and dystrophic calcifications are formed, often bilateral and symmetrical. Suppose the serum levels of calcium and phosphorus are within normal limits. In that case, calcification in normal soft tissue is idiopathic, and calcification in normal serum levels in dead and degenerated tissue is called dystrophic calcification [1]. These calcifications can be found in various soft tissues. Carotid artery calcifications are important causes of embolism and cerebrovascular occlusion. They are observed as radiopaque lesions near the third and fourth cervical vertebrae and above or below the greater horn of the hyoid bone. These calcifications are usually numerous, with clear boundaries and vertical structures [2, 3]. Pulp stones could be free or attached to the dentin. They are often round and oval, but they can also be irregular. Pulpal stones are radiopaque findings that may be found in a pulp chamber, root canal, or both [4, 5]. Aging, orthodontic forces, periodontal diseases, fluoride intake, systemic diseases, long-term exposure to aggressive factors such as caries, deep restorations, moderate wear, and some genetic defects increase the incidence of pulp stones [6, 7]. The complications of diabetes, such as end arthritis, hypoxia in mature teeth, and increased inflammatory response in patients with chronic diabetes, play a vital role in producing pulp stones [7]. The prevalence of pulp stones was reported to be higher in women, maxillary teeth, and first molars [8–10]. Another study around the globe has mentioned the prevalence of 10 to 20% in all permanent teeth [9]. The prevalence of pulp stones in the Iranian population is 31.5%, which is more commonly observed in women. The probability of the incidence of pulp stone exists in 9.6% of all permanent teeth [9]. Diabetes, as a common metabolic disease, has extensive effects on the body [11]. In a retrospective study in Saudi Arabia, the prevalence of pulp stones was assessed in CBCT views. They evaluated 179 CBCT (73 patients with cardiovascular diseases and 76 with type I and II diabetes). Also, 80 healthy controls have entered the study [12]. Pulp stones were significantly observed more in cardiovascular diseases over the age of 50. There were a significant number of pulp stones in the first maxillary molars of cardiovascular patients. The findings revealed that the presence of pulp stones in cardiovascular and diabetic patients was 2.94 times that of the healthy controls [12]. Khambet et al. (2021) showed that atherosclerotic plaques were observed in 26% of the panoramic views of diabetic patients [13]. This finding was confirmed by the authors [12, 14, 15].

The risk of affliction with heart disease in diabetic patients with carotid artery calcification is similar to people with a history of coronary disease. Thus, it is important to diagnose carotid artery calcification in diabetic patients [16]. Discovering the pulp stones and carotid artery calcification on a panoramic view could indicate systemic diseases [17]. This study aimed to investigate the relative frequency of carotid calcifications and pulp stones in panoramic radiographs of diabetic patients.

## Research gap and novelty statement

### Lack of prior research

No studies have specifically evaluated the simultaneous presence of pulp stones and carotid artery calcifications in diabetic patients. Given that both conditions may share underlying pathophysiological mechanisms related to vascular and metabolic disturbances, understanding their correlation could provide valuable insights into early diagnostic markers for diabetes-related complications. Furthermore, this study uniquely assesses these variables based on HbA1c levels, offering a direct link between glycemic control and calcification patterns. This highlights a significant gap in the literature and underscores the need for further exploration of these potential early indicators in diabetic patients.

### Novelty of the present study

This study addresses the lack of research on pulp stones and carotid artery calcifications in diabetic patients, a finding that has been largely ignored in previous studies. Examining this population closely provides valuable insights into their unique oral health needs and challenges.

## Materials and methods

### Study population

This retrospective observational study was carried out from December 2021 to February 2022. The study participants included 107 diabetic patients referred to Yazd Dental School who required panoramic radiography after dental examination. They should have been between 18 and 69 years old and had recently been visited by an endocrinologist. All methods were conducted per relevant guidelines and regulations. We reported our findings according to the STROBE guidelines.

To ensure the highest standards of ethical conduct, rigorous measures were implemented to protect participant confidentiality and data security. All participant data were anonymized and stored securely on password-protected servers with restricted access. Additionally, physical copies of data were kept confidential. Patients were informed about the study's aim, and informed consent was obtained from each patient before initiating the study. All methods were performed according to the relevant guidelines and regulations.

The study was carried out per the Declaration of Helsinki guidance of the World Medical Association and Good Clinical Practice recommendations. The Ethics Committee of Research Shahid Sadoughi University of Medical Sciences, Yazd (IR.SSU.DENTISTRY.REC.1400.013) approved all the experimental procedures in the present study.

### Statistical analysis

The sample volume was determined based on a similar study. [18], which stated the frequency of carotid calcifications to be 37% (*P* = 37%), with a confidence interval of 95% (α = 0.5), and accuracy of 0.09 (d = 0.09). The first group included 107 diabetic patients referred to the Yazd Faculty of Dentistry for dental services, who had an HbA1C test in the last three months, and who needed a panoramic radiograph after oral and dental examination. They were selected by a convenience method. To increase confidence, the second group consisted of 300 healthy people who came to the Yazd Dental Faculty for dental services and needed a panoramic radiograph after oral and dental examination. While the larger control group provides a more representative sample of the general population, we acknowledge the potential impact on statistical power. Both groups were matched in terms of age and gender. Using a random numbers table, the samples were randomly selected from the statistical population.

$${{n}}=\frac{{{{Z}^{2}_{1-\frac{a}{2}}}}p(1-p)}{d^{2}}$$

All patients gave informed written consent before radiography. There was no statistically significant difference between the two groups in terms of age and gender (*P* > 0.05).

The patient's status was evaluated (based on the recent HbA1c in the patient's file) and recorded in the relevant form.

### Data collection

The patients' ID was recorded in a special form after the panoramic examination. This study used Plan Meca Promax 2d S3, Scara3 panoramic device (66 kVp, 6 mA, 18 s, made in Finland) to perform panoramic radiography. The inclusion criteria are being between 18 and 69 years old, the absence of confirmed systemic diseases such as ischemic heart disease or renal diseases, and having at least 4 posterior teeth (except the third molar). It is important to note that all individuals referred to the Oral and Maxillofacial department have a complete systemic file and undergo thorough screening. Those exhibiting any suspicious symptoms will not be included in the study. Also, they should not be smokers, too.

Exclusion criteria regarding the quality of panoramic X-rays were considered. Low-quality panoramic views, in which it was not possible to distinguish details, were excluded from the study. Detected pulp stones should belong to the vital premolars and molars, without indirect and big restorations or history of orthodontic treatment, with non-obliterated pulp chambers. Teeth with root canal therapy, root resorption, and unerupted teeth were excluded. The exclusion criteria regarding risk factors of pulp stones (age, orthodontic treatment) were considered.

Unilateral or bilateral calcifications in the projection of carotids, radiologically suspicious for carotid artery calcifications, have been detected [19–21].

**Fig. 1:**
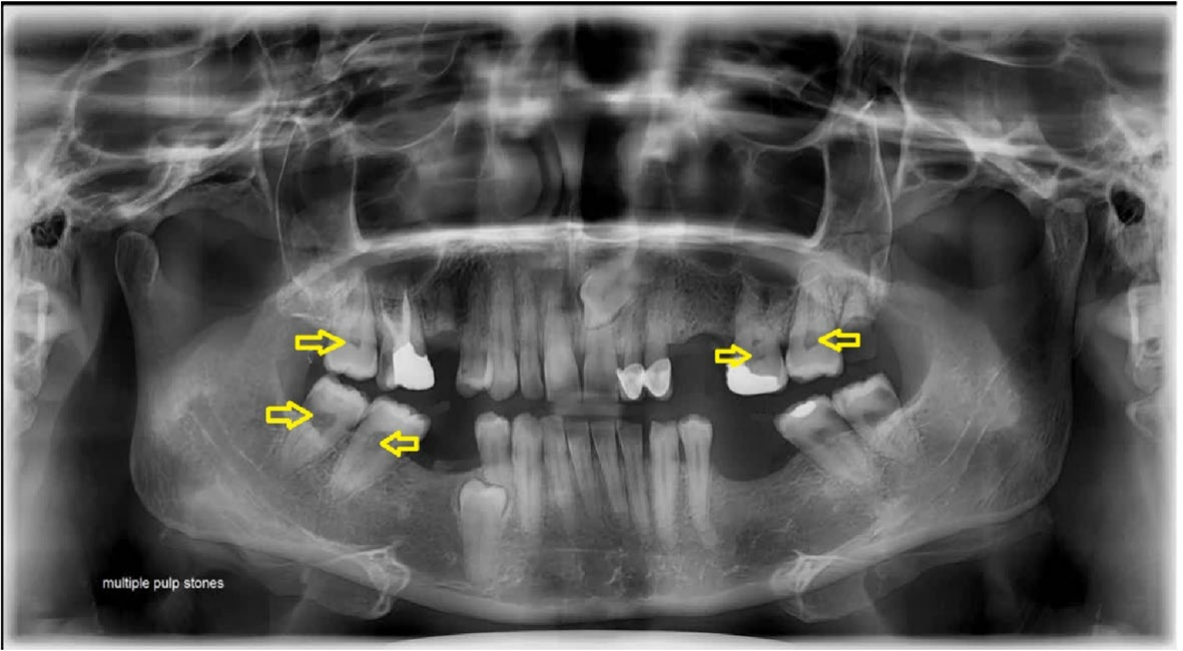
Multiple pulp stones

**Fig. 2:**
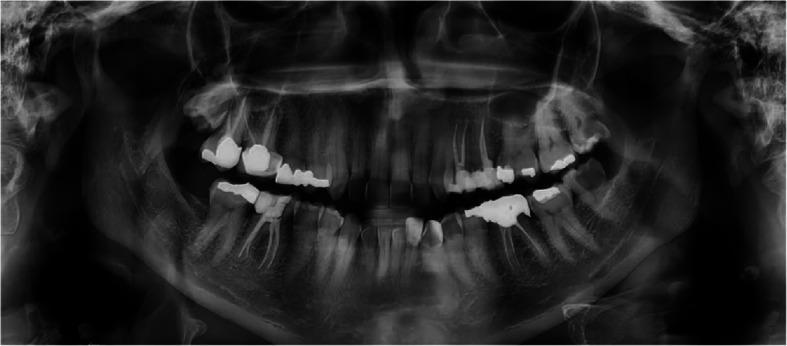
Carotid artery calcifications

Digital panoramic radiographs revealed pulp stones identified as one or multiple radiopaque masses in the pulp chamber of both maxillary and mandibular premolars and molars (Fig. 1). Additionally, carotid artery calcifications appeared as nodular, punctate, or vertico-linear radiopacities in the soft tissues of the neck, positioned posteroinferior to the angle of the mandible, at the level of the lower margins of the C3 and C4 vertebrae, and adjacent to the epiglottis and hyoid bone, either above or below these structures [22](Fig. 2). In addition to carotid calcifications and pulp stones, the total number of teeth and the number of teeth involved, except the third molar teeth, were evaluated.

An educated last-year dental student evaluated all evaluations. It was done using a magnifying glass on a single negatoscope under the supervision of an oral and maxillofacial radiologist. She was masked about the participants. To avoid selection bias, the radiographs were numbered (Table 1).

**Table 1:** Demographic characteristics of the study samples

Study samples	Male N (%)	Female N (%)	25–49 years N (%)	50–64 years N (%)	Total
Diabetic patients	40(62.6)	67(62.6)	42 (44.7)	65 (55.3)	107(100)
Healthy persons	104(34.7)	196(65.3)	147 (49)	153 (51)	300 (100)

## Results

**Fig. 3:**
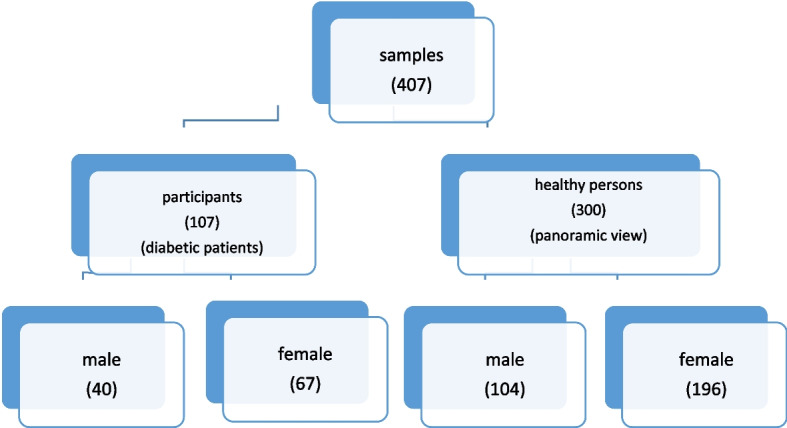
Flowchart of the study samples

No patients were excluded, as we ensured that all participants met the predefined inclusion criteria. There was no specific exclusion criterion applied, and all eligible patients were included in the analysis. Figure 3 shows the flowchart of the study population. The samples of this retrospective observational study were evaluated. They were in the 25 to 64-year-old age group, with a mean age of 49.7 years. The frequency of carotid artery calcification was 42 (14%) in healthy persons and 44 (41.1%) in diabetic patients. There was a significant difference between the two groups. Pulp stones were observed in 41 (38.3%) diabetic patients and 64 (21.3%) healthy persons. Diabetics had significantly more pulp stones than others (Table 2).

**Table 2:** The frequency of carotid artery calcification and pulp stones in the studied groups

Study samples	Diabetic patients N (%)	Healthy persons N (%)	*P* value
Carotid artery calcification			
Present	44 (41.1)	42 (14)	0.0001
Absent	63 (58.9)	258 (86)	
Pulp stones			
Present	41 (38.3)	64 (21.3)	0.001
Absent	66 (61.7)	236 (78.7)	
Total	107 (100)	300 (100)	

Chi-square test

The chi-square test showed that carotid artery calcification and pulp stone were significantly higher in diabetic patients in terms of gender and age groups (*P* < 0.05) (Tables 3 and 4). Moreover, the relative frequency of unilateral and bilateral carotid artery calcification in diabetic patients was significantly higher than in healthy subjects (*P* < 0.05) (Table 5).

**Table 3:** Frequency of carotid artery calcifications and pulp stones in terms of gender and age groups

Groups	Variables	Status of Carotid Artery Calcifications			Status of Pulp stones		
		Present N (%)	Absent N (%)	*P*-Value	Present N (%)	Absent N (%)	*P*-Value
Gender	Male						
	Diabetic patients	13(32/5%)	27(67.5%)	0.003	13(32.5%)	27(67.5%)	0.033
	Healthy persons	12(11.5%)	92(88.5)		17(16.3%)	87(83.7%)	
	Female						
	Diabetic patients	31(46.3%)	36(53.7%)	0.0001	28(41.8%)	39(58.2%)	0.005
	Healthy persons	30(15.3%)	166(84.7%)		47(24%)	149(76%)	
Age groups	25-49						
	Diabetic patients	14(33.3%)	28(66.7%)	0.003	18(42.9%)	24(57.1%)	0.012
	Healthy persons	320(13.6%)	35(53.8%)		34(23.1%)	113(76.9%)	
	50-64						
	Diabetic patients	30(46.2%)	35(53.8%)	0.0001	23(35.4%)	42(64.6%)	0.013
	Healthy persons	22(14/4%)	131(85/6%)		30(19.6%)	123(80.4%)	

chi-square test

**Table 4:** Frequency of carotid artery calcifications and pulp stones  based on healthy condition

Groups	Variables	Status of carotid artery calcifications			Status of Pulp stones		
		Present N (%)	Absent N (%)	*P*-Value	Present N (%)	Absent N (%)	*P*-Value
Diabetic patients	Gender						
	Male	13(32/5%)	27(67/5%)		13(32.5%)	27(67.5%)	0.339
	Female	31(46/3%)	36(53/7%)	0.339	28(41.8%)	39(58.2%)	
	Age groups						
	25-49	14(33/3%)	28(66/7%)		18(42.9%)	24(57.1%)	0.438
	50-64	30(46/2%)	35(53/8%)	0.188	23(35.4%)	42(64.6%)	
Healthy persons	Gender						
	Male	12(11/5%)	92(88/5%)		17(16.3%)	87(83.7%)	0.125
	Female	30(15/3%)	166(84/7%)	0.125	47(24%)	149(76%)	
	Age groups						
	25-49	20(13/6%)	127(86/4%)		34(23.1%)	113(76.9%)	0.457
	50-64	22(14/4%)	131(85/6%)	0.847	30(19.6%)	123(80.4%)	

chi-square test

**Table 5:** Frequency of carotid artery calcifications and pulp stones in terms of HbA1c in diabetic patients

	HbA1C<7 N (%)	HbA1C>7 N (%)	*P*-Value
Status of carotid artery calcifications			
Unilateral	14(17.3%)	6(23.1%)	0.571
Bilateral	17(21%)	7(26.9%)	
Absent	50(61.7%)	13(50%)	
Status of Pulp stones			
Present	27(33.3%)	14(53.8%)	0.061
Absent	54(66.7%)	12(46.2%)	

Chi-square test

**Fig. 4:**
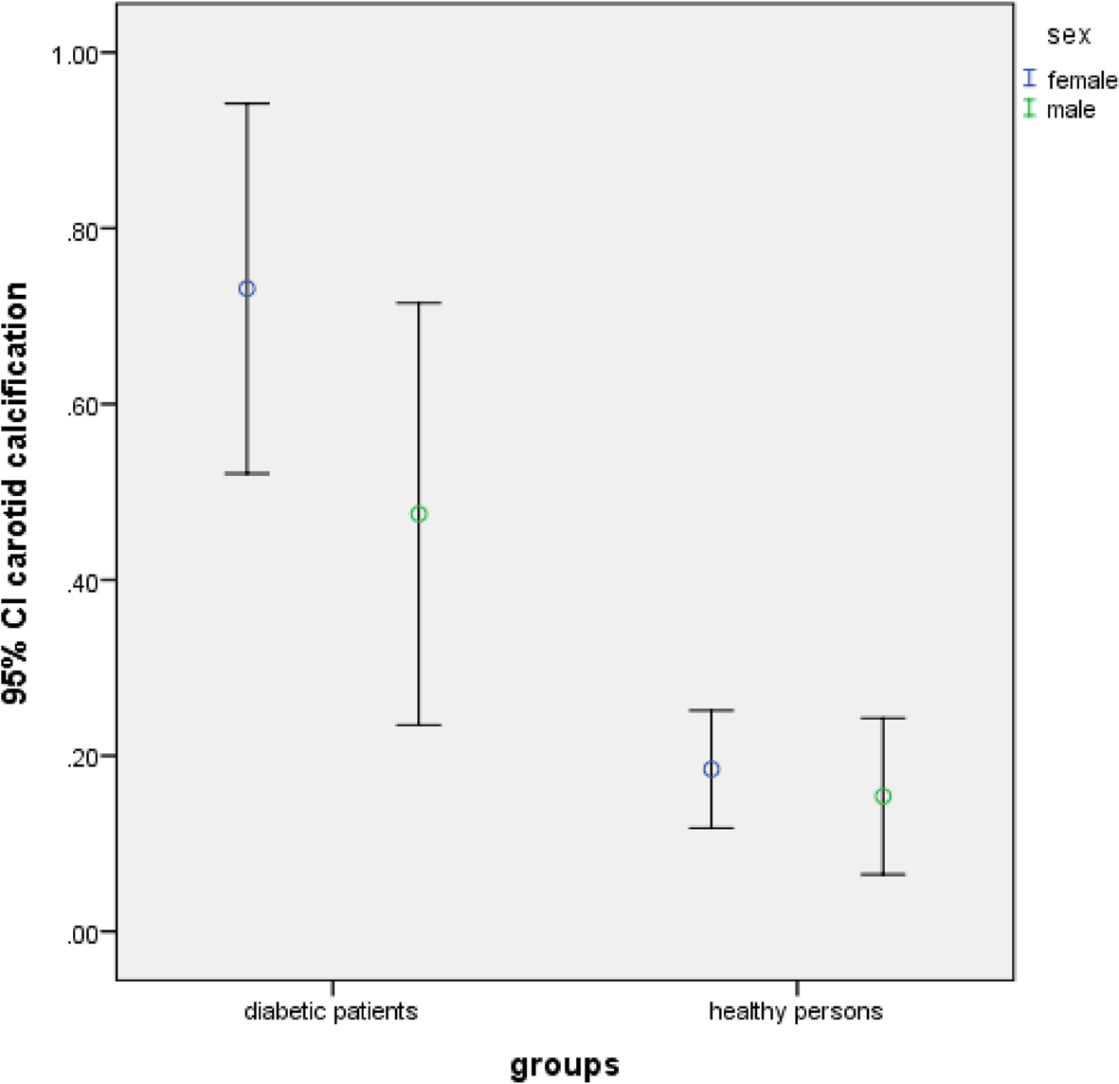
Frequency of carotid artery calcifications in participants based on gender (*P*-value = 0.339 in diabetic group, *P*-value = 0.125 in healthy persons)

**Fig. 5:**
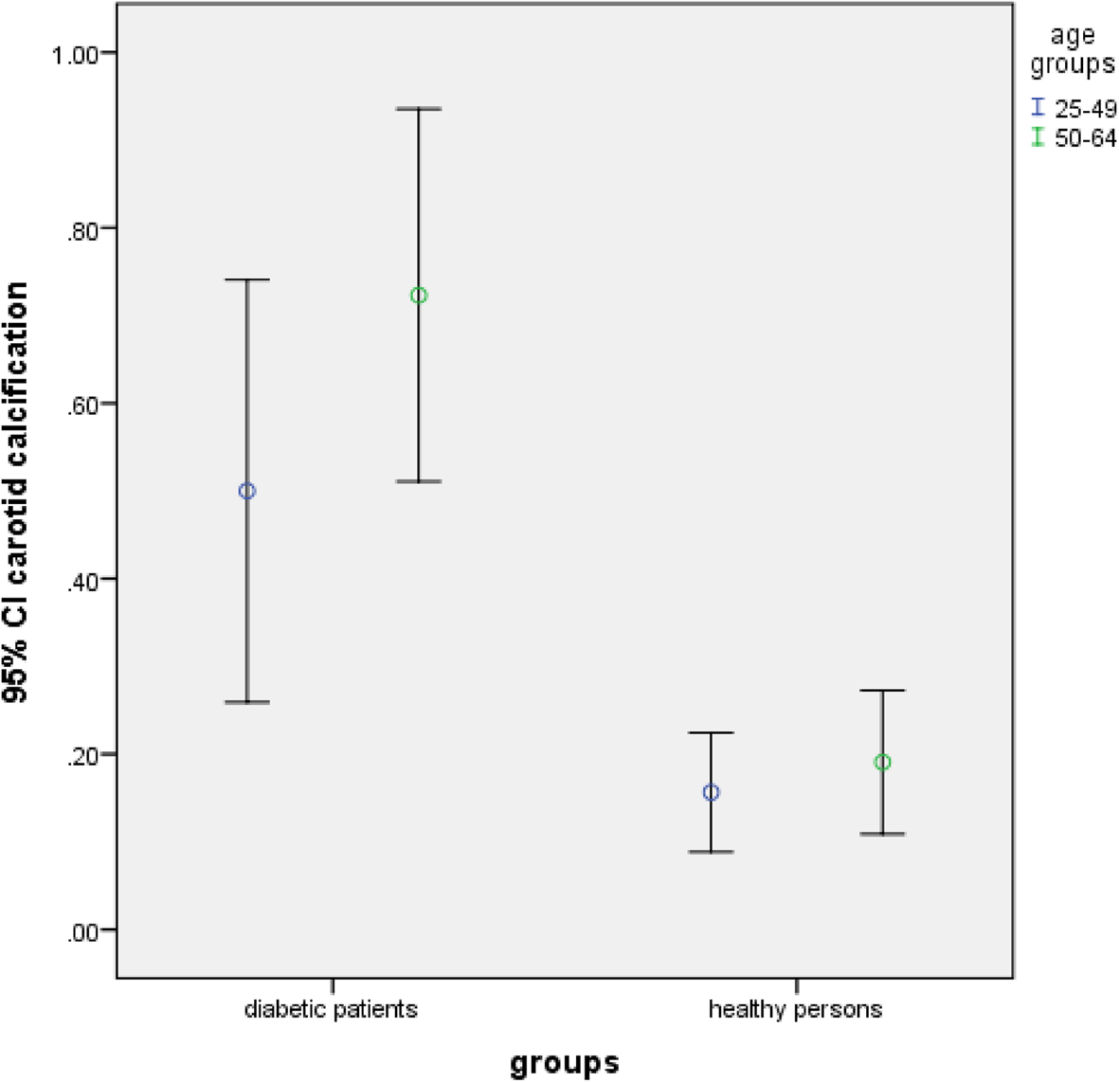
Frequency of carotid artery calcifications in participants based on age groups (*P*-value = 0.188 in diabetic group, *P*-value = 0.847 in healthy persons)

**Fig. 6:**
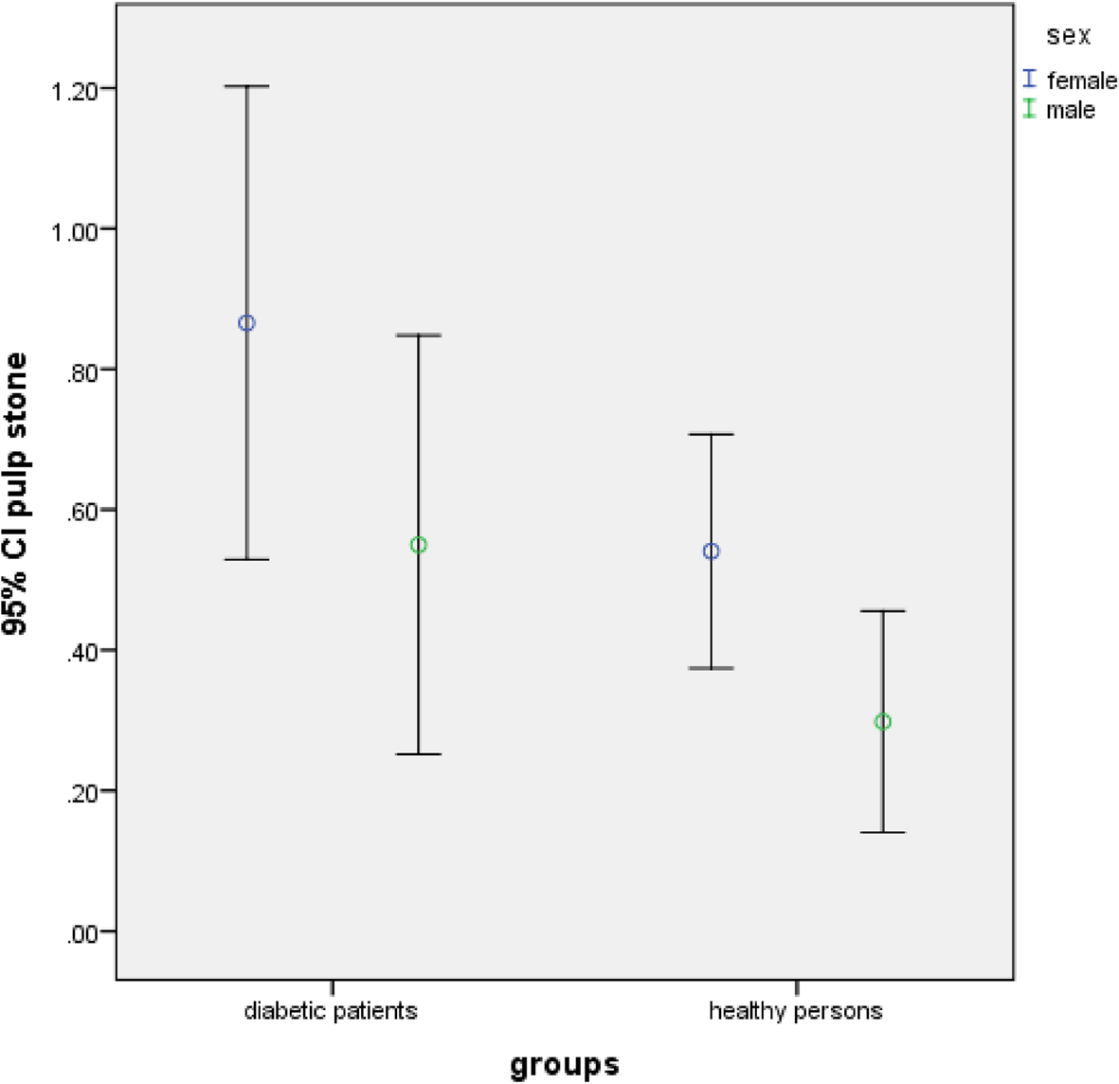
Frequency of pulp stone in participants based on gender (*P*-value = 0.339 in diabetic group, *P*-value = 0.125 in healthy persons)

**Fig. 7:**
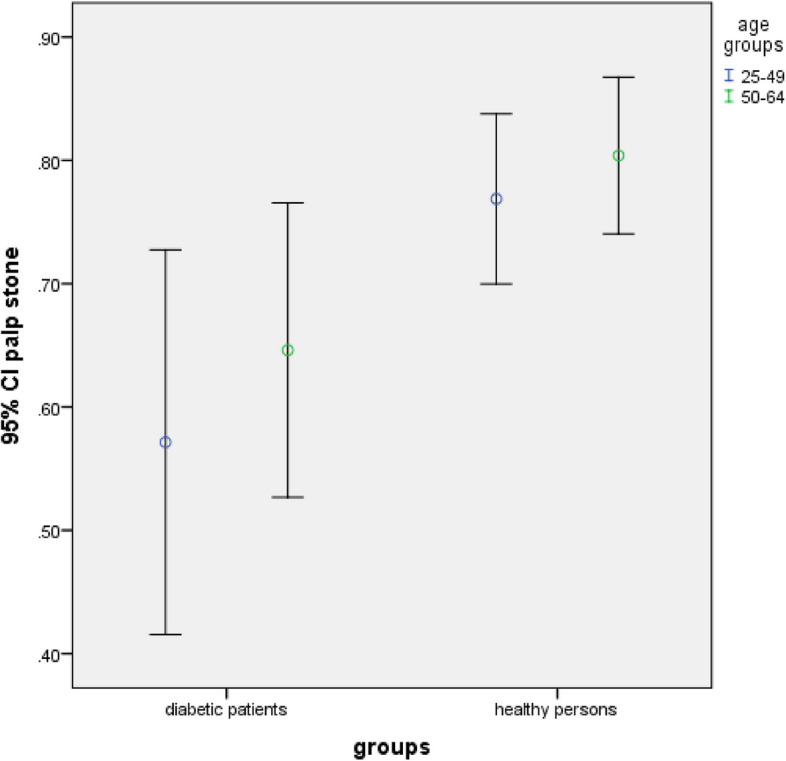
Frequency of pulp stone in participants based on age groups (*P*-value = 0.438 in diabetic group, *P*-value = 0.457 in healthy persons)

There was no significant difference in the frequency of carotid artery calcification and pulp stone between the studied groups regarding age groups and gender (*P* > 0.05) (Table 4 and Figs. 4, 5, 6 and 7).

Additionally, the relative frequency of carotid artery calcification and pulp stone in the diabetic group was not significant in terms of HbA1c (*P* > 0.05). As can be seen in Table 5, there was no significant difference in the frequency of pulp stones and carotid artery calcification between the two diabetic groups according to HbA1c. So, the frequency of pulp stones and carotid artery calcification was similar in controlled and uncontrolled diabetic patients.

The highest number of pulp stones was also observed in maxillary second molars**.** The Relative Risk (RR) of pulp stones in diabetic patients compared to healthy persons was 1.79; that is, the risk of pulp stones in the diabetic group was 1.79 times higher than that of healthy persons.

With a confidence interval of 95%, the relative risk obtained ranged from 1.3 to 2.46, which was statistically significant (*P* < 0.05). The relative risk of carotid artery calcification in diabetic patients was 2.60 compared to that of healthy people. This means that the risk of carotid artery calcification in the diabetic group was 2.60 times that of healthy persons. With a confidence interval of 95%, the relative risk obtained ranged from 1.92 to 3.52, which was statistically significant (*P* < 0.05). Based on the findings, carotid artery calcification is more important as a predictor of diabetes mellitus.

Finally, the relative risk of carotid artery calcification in patients with pulp stones was 1.56. With a confidence interval of 95%, the relative risk obtained ranged from 1.11 to 2.21, which was statistically significant (*P* < 0.05).

## Discussion

In this study, the frequency of carotid calcification in diabetic patients was significantly higher than in healthy subjects; it was consistent with some previous studies [13–15, 23]. In a Swedish study, it was shown that subclinical atherosclerosis in the coronary arteries was more common in people with diabetes and prediabetes compared to normoglycemic participants [24]. They evaluated 50–64 years of ultrasound examination, while the age range in this study was 25–64 years [24]. Complications of diabetes, such as endothelial dysfunction, changes in mineral metabolism, and increased production of inflammatory cytokines that can cause atherosclerotic calcifications, were expected [25].

Pulp stones, often considered a defensive response of the dental pulp to chronic irritation and inflammation, exhibited a heightened calcification in diabetic individuals. This increased calcification was accompanied by elevated levels of osteopontin and alkaline phosphatase activity, suggesting enhanced mineral deposition and bone-like tissue formation within the pulp [26]. A histologic study suggested that type 2 diabetes may show a similar response in the pulp to complications in other body sites. Hyperglycemia led to morphology changes of the clinically normal dental pulp with altered immune cell and cytokine expression [27]. The osteoblastic process typically initiates pulp calcification activity. This process is characterized by the formation of an osteoid matrix, which is deposited by odontoblast cells in the peripheral regions of the pulp. Consequently, this matrix acts as a triggering factor for the onset of calcification in the pulp, ultimately leading to pulpal obliteration [28]. Side effects of diabetes, such as atherosclerosis, poor blood circulation in adult teeth, and increased inflammatory response in patients with chronic diabetes, contribute to the development of pulp stones [12].

There is evidence that high triglycerides in diabetic patients, mediated by inflammatory cytokines, may play an important role in carotid calcification [29]. In the current study, the frequency of carotid calcifications and pulp stones was not significantly different in terms of age; this finding was confirmed by some previous studies [12, 14, 23]. Nonetheless, in one study, the highest prevalence of carotid calcification was related to the age group of over 70 years [13]. It could be justified by the aging process. It may be due to the aging process. To reduce the impact of aging on vascular calcifications in this study, participants were restricted to those under 70 years old. The incidence of pulpal calcification increases with age. Additionally, another study found that the frequency of pulp stones in the 31 to 45 age group was significantly higher than in other age groups, likely because they had more teeth available for examination [7]. The frequency of carotid artery calcification and pulp stones was not significantly different in terms of gender, either. It went in line with some previous studies [12, 14, 15, 23]. In one study, a slightly higher prevalence of carotid calcification in men was attributed to more smoking and alcohol abuse in men. Presumably, the protective effect of estrogen and progesterone against thromboembolic events would be expected [13]. Nevertheless, some studies suggest that the frequency of pulp stones may be higher in men, potentially due to greater occlusal forces and trauma-related factors. However, variations in reported findings could stem from multiple influences, including systemic conditions, dental history, genetic predispositions, and methodological differences across studies. Factors such as age range, orthodontic forces, periodontal diseases, fluoride exposure, deep restorations, and ethnic diversity may all contribute to discrepancies in prevalence rates. While our study identifies an association, further research is warranted to clarify the underlying mechanisms influencing pulp stone formation in different populations [6].

Alamoudi and colleagues investigated the relationship between pulp stones, pulp calcifications, and systemic diseases. Their results, unlike those of the present study, did not show a significant difference in the presence of pulp calcifications between healthy individuals and those with systemic diseases [30]. The discrepancy between the two studies may be attributed to the overall assessment of systemic disease in the participants. In this study, only diabetic patients were examined. Romano et al. suggested a link between kidney stones and pulp stones, while Khan et al. reported a statistically significant association between diabetes and pulp stones [31, 32].

In the present study, the frequency of carotid calcification and pulp stones in terms of HbA1c was not significantly different; this was consistent with some previous studies [14, 23]. Menegazzo demonstrated significant differences between diabetic and non-diabetic groups in plaque calcium content, tissue composition, and gene expression linked to instability, inflammation, and calcification [33].

One of the advantages of the present study was the simultaneous evaluation of the relative frequency of carotid calcification and pulp stones in diabetic patients compared to healthy persons. This point was ignored in the past. The relative risk of carotid calcification and pulp stones in diabetic patients was 2.93 times and 1.8 times that of the healthy group, which was statistically significant. In another study, the risk of the presence of pulp stones in diabetic patients was 1.81; this was consistent with the present study [12]. This finding was compatible with another CBCT-based study that was done in Saudi Arabia. Diabetic patients were found to have 1.81 times (*p* < 0.01; CI 0.48–2.06) higher risk of having pulp stones in comparison to healthy subjects [12]. Furthermore, the relative risk of carotid calcification in patients with pulp stones was 1.56. With a confidence interval of 95%, the relative risk obtained ranged from 1.11 to 2.21, which was statistically significant; yet, this was not investigated in other studies.

In this study, the frequency of pulp stones in diabetic patients was significantly higher than in healthy persons; this was consistent with some previous studies [7, 12]. Microvascular complications of diabetes could have an important role [12, 34].

The results of the present study indicate that diabetic patients exhibited a higher prevalence of carotid artery calcifications and pulp stones compared to healthy individuals. While this observation suggests a potential link, it does not establish a direct predictive relationship. Given the presence of unknown parameters and possible confounding factors, further research with larger sample sizes and rigorous methodological controls is required to better understand the clinical significance of these findings.

This study has several limitations. The imbalanced sample size may affect generalizability, and the lack of lipid profile data and other confounders limits further analysis. Given the observational nature of the study, causality cannot be established, and validation in larger, more diverse populations is needed. Additionally, diabetes duration and medication use were not considered, which could impact vascular and dental calcifications. Future studies should stratify diabetic patients, account for treatment regimens, and use longitudinal designs to clarify these associations.

## Conclusion

This cross-sectional study suggests a possible association between diabetes mellitus and the prevalence of carotid artery calcifications and pulp stones. However, given the observational design and potential confounding factors, these findings should be interpreted with caution. Further prospective studies with larger sample sizes and stronger methodological controls are needed to determine the nature of this relationship more conclusively. Until more definitive evidence is available, clinicians should continue to focus on comprehensive risk assessment and management of cardiovascular health in diabetic patients.

## Data Availability

The datasets analyzed during the current study are available from the corresponding author upon reasonable request.
